# Intelligence Assessment of Children & Youth Benefiting from Psychological-Educational Support System in Poland

**DOI:** 10.1038/s41597-024-03663-9

**Published:** 2024-07-27

**Authors:** Michał Olech, Paweł Jurek, Bartosz M. Radtke, Urszula Sajewicz-Radtke, Ariadna Łada-Maśko

**Affiliations:** 1 Medical University of Gdańsk, Gdansk, Poland; 2 University of Gdansk, Gdansk, Poland; 3 Laboratory of Psychological and Educational Tests, Gdansk, Poland

**Keywords:** Patient education, Population screening

## Abstract

This article presents a unique dataset comprising 419,135 intelligence assessment results. The study utilised the Polish adaptation of the Stanford-Binet Intelligence Scale 5 during individual diagnostic sessions conducted under natural conditions. The research included children aged 3;0–18;11 of both genders who had been referred to support institutions (psychological-educational counselling centres, post-hospital clinics, hospital departments) by preschools or schools, or voluntarily requested by parents with their consent. The data collection spanned the entire country of Poland from 2018 to 2023. In addition to comprehensive intelligence assessment results, the dataset contains valuable demographic information, enabling in-depth analyses. The dataset’s uniqueness lies in its impressive sample size, encompassing over four hundred thousand observations as well as the utilisation of time-consuming and thorough intelligence assessment procedures in settings that mimic the real world. Moreover, the context of the study is noteworthy, as the participants are individuals benefiting from the publicly - available Polish psychological support system.

## Background & Summary

Assessing intelligence levels has a lengthy and rich tradition. Since the first scale was created – the Binet-Simon scale in 1905 – up to today, its strong relationship with academic achievements^1–3^ and its ability to predict success in higher education^4^ have always been highlighted. Moreover, it is a key factor in procedures for diagnosing neurodevelopmental disorders, such as intellectual development disorders, developmental learning disorder, and autism spectrum disorders^5,6^. In addition to this, diagnosing levels of intellectual ability provides an important part of the clinical picture of other conditions and disorders, for both mental and somatic health^5,6^. Reliable and accurate assessment of intelligence quotient (IQ) levels can help identify appropriate therapy and education programmes for a given patient. Results of IQ measurement are also used to select students for special education programmes – both in the context of intellectual disability and gifted students^7–9^.

There are currently many tests that measure various aspects of intelligence, which is an extremely complex construct^10^. The theory of Cattell-Horn-Carroll (CHC)^11^ provides a model of the structure of intelligence that is commonly used in research. All contemporary intelligence tests are based on its assumptions^12^.

The choice of the appropriate diagnostic method depends on many factors, including age, language proficiency, motor skills, and potential disabilities. The process of diagnosing such a complex construct as intelligence in children and adolescents is significantly influenced by motivational factors. Therefore, assessment procedures, especially for younger children, often incorporate play and activities to enhance engagement and motivation during the diagnostic process. For a detailed description of the most commonly used methods for assessing intelligence in children and adolescents, refer to the work by Flanagan and McDonough^13^.

Some intelligence tests measure a narrow range of selected intellectual skills. In order to achieve the widest possible picture of a person’s intellectual functioning when diagnosing children and youths, complex test batteries are used, such as the Stanford Binet Intelligence Scales – 5^th^ edition (SB5)^14,15^, the Wechsler Preschool and Primary Scale of Intelligence - Fourth Edition (WPPSI-IV)^16^ and the Wechsler Intelligence Scale for Children – Fifth Edition (WISC-V)^17^. There exist researchers who believe that the WISC-V scales should be treated as a screening tool^2^, while the SB5 should be used to test special groups^18^, who are the main recipients of psychological help. Table 1 compares the range of the intelligence factors measured by those two tools, in the context of the CHC theory.

**Table 1:** Selected factors of the second stratum in the CHC theory together with a short description of characteristics and information about the ranges measured by the so-called ‘big’ intelligence scales.

FACTOR IN THE CHC THEORY	DESCRIPTION	ASPECT	SB5	WISC V
Fluid Reasoning	The ability to reason, solve problems requiring understanding relationships and dependencies, innate intellectual potential.	non-verbal	x	x
		verbal	x	—
Crystallized Intelligence	Knowledge and skills acquired through education and experience.	non-verbal	x	—
		verbal	x	x
Visual-spatial Processing	The ability to spot patterns and dependencies in visual material, visual analysis and synthesis, and understanding spatial relationships.	non-verbal	x	x
		verbal	x	—
Short-term Memory	The process of registering, temporarily storing, processing, and organising information in memory.	non-verbal	x	x
		verbal	x	x
Quantitative Knowledge	Mathematical reasoning, including, e.g., understanding the concept of numbers, estimation, solving tasks.	non-verbal	x	—^1^
		verbal	x	—
Processing Speed	The speed of information processing, the time needed for an appropriate reaction.	non-verbal	—	—
		verbal	—	x
		verbal	—	x

Based on: Grégoire^38^, Wechsler^17^, Roid, Sajewicz-Radtke, Radtke, Lipowska^15^.

^1^Quantitative reasoning emerges in a set of so-called auxiliary indices that are not included in the factors of the WISC-V intelligence measure^17,39^. After: Radtke & Sajewicz-Radtke^40^.

In institutions that make up the psychological-educational support system in Poland, the SB5 test battery, which has norms and testing procedures for children as young as two years old, is commonly used for assessing intelligence in children and adolescents. Administering the SB5 requires not only a diploma in psychology, but also additional training and practice^15^, making it costly and time- and labor-intensive to use. Despite these challenges, intelligence assessment plays a crucial role in designing targeted diagnostic and therapeutic interventions for patients and clients, making it widely utilized in psychological-educational counseling centers and health centers^19^. However, both in Poland and globally, intelligence assessment primarily occurs through individual patient assessments, lacking the collective data aggregation needed for developing comprehensive systemic support. This approach contrasts with emerging trends in clinical psychology, which emphasize integrating psychological diagnosis with intervention^20^. Furthermore, research on intelligence published to date consists of rather small clinical trials, usually involving hundreds of participants^21–27^. Most such publications concern research using intelligence screening tools or quick non-verbal ones (e.g., Raven’s Progressive Matrices^28^, Cattell’s Fluid Intelligence Test^29^)^25,30–33^, which unfortunately do not take into account the complex structure of intellect and are much less comprehensive and multidimensional than intelligence test batteries, such as the above-mentioned SB-5^14,15^ and WISC-V^17^.

Notably, as far as we are aware, there are no existing available datasets that contain data that constitute a comprehensive intelligence assessment (full IQ, non-verbal IQ, verbal IQ, fluid reasoning, knowledge, quantitative reasoning, visual-spatial reasoning, working memory), along with demographic variables such as sex, parents’ education level and place of residence of children who are recipients of public mental health services.

The dataset described in the current paper offers immense potential for a variety of research questions and analyses. Researchers can explore the intricate relationships between demographic variables and intelligence quotients (IQ), unveiling how factors such as age and gender associate with cognitive abilities. Latent profile analyses can be conducted to identify cognitive ability profiles among individuals with intellectual disabilities, providing insights into the diverse nature of cognitive impairments. The dataset also allows for a comprehensive examination of the factor structure of the SB5 tests, facilitating the validation and refinement of this widely-used assessment tool in the Polish context. Moreover, measurement invariance (MI) can be assessed across different groups, such as gender and residence, ensuring that the SB5 measures intelligence consistently across these subgroups.

The application of machine learning methods to this dataset can further enhance our understanding of learning difficulties and developmental disorders. By modeling the occurrence of these challenges, researchers can develop predictive tools and intervention strategies that are grounded in extensive empirical evidence. The unique and large sample size, coupled with the naturalistic assessment conditions, ensures that findings derived from this dataset are both robust and generalizable to real-world settings. The sheer scale and comprehensiveness of this dataset make it a valuable resource for advancing the field of psychological assessment and educational support, enabling researchers to address critical questions and develop evidence-based practices that can significantly benefit individuals within the public psychological support system in Poland and beyond.

## Methods

This study was conducted in accordance with the principles of the Declaration of Helsinki. Approval was granted by the Ethics Board for Research Projects at the Faculty of Social Sciences, University of Gdansk, Poland (decision no. 13/2022). The Ethics Board also adheres to the principles set forth in Regulation (EU) 2016/679 of the European Parliament and of the Council of 27 April 2016 on the protection of natural persons with regard to the processing of personal data and the free movement of such data, and repealing Directive 95/46/EC (General Data Protection Regulation, GDPR). This regulation further allows for the open publication of data when the requisite criteria are met. The Ethics Board does not impose stricter licensing requirements for this dataset and has authorized its open publication under a CC-BY license. Additionally, our research plan has already been published in the Open Science Framework repository^34^. Furthermore, the parents or guardians of the children involved provided written informed consent for the use of the data in research. Moreover, when entering demographic data and raw test results into the online application, the diagnostician also confirmed consent for the open publication of the data.

Participants were children of both genders aged 3;0–18;11 who had been referred to clinics for support (psychological–educational clinics, hospital clinics, hospital wards) by their kindergartens or schools, or at the request of their parents (always with parental consent). The research procedures were administered throughout Poland between 2018 and 2023. All participants used the Polish language sufficiently well to allow them to understand the instructions and tasks as well as to answer questions.

The study used the Polish adaptation of the Stanford Binet Intelligence Scales – 5^th^ edition (SB5)^15^ administered during an individual diagnostic session. The SB5 scale is a standard IQ scale that measures five broad intellectual skills: Fluid Reasoning, Knowledge, Quantitative Reasoning, Visual-Spatial Processing, and Working Memory. Each of these factors is measured in two aspects – verbal and non-verbal – giving a total of 10 factors that cover a wide spectrum of the structure of intelligence. The scale has many potential applications, including diagnosing developmental disorders, clinical and neuropsychological assessments, psycho-educational diagnostics in the context of learning disabilities and special education, assessing levels of intellectual disability, and qualifying students to special educational programmes for particularly gifted individuals. However, it is important to note that assessing intellectual functioning is not the only factor to consider in the diagnostic process. Both ICD-11^35^ and DSM-5^36^ emphasize the importance of incorporating adaptive behavior into the comprehensive assessment of disabilities. ICD-11^35^ underscores the use of adaptive behavior assessment as a critical tool for clinicians to evaluate the severity of intellectual developmental disorders (mild, moderate, severe, or profound). This assessment can complement intelligence tests or serve as an alternative when standard measures are not feasible due to factors such as the individual’s cultural or linguistic background.

The measurement includes performing a range of varied tasks, such as defining words, solving tasks and mathematical and logical problems, building patterns from blocks based on the images shown, and recalling words and visuo-motor sequences.

The scale was normalised on a country-wide representative sample of 3,246 individuals. The results of the SB5 IQ test are characterised by high reliability for the full scale as well as the non-verbal IQ, verbal IQ, and short versions of the scale (0.92–0.98). The average reliability of the five factors is between 0.88–0.91, and of the 10 factors between 0.78–0.89^37^.

The presented data significantly differ from those collected during the normalization and standardization process. While the normalization data reflect the characteristics of a population consisting of healthy individuals without significant developmental difficulties, the described data pertain to a specific group of public health service recipients reported by caregivers due to experiencing various types of developmental problems. Additionally, the normalization group included adults over the age of 18, whereas the described database contains only data regarding children and adolescents.

Most commonly, the first step in diagnostic procedures for children referred to the mental health care system is to assess for potential intellectual disabilities. This initial evaluation typically includes measuring IQ levels, with the SB5 test being widely used for this purpose in Poland. Clinicians originally utilized the data from the dataset in these diagnostic processes.

The SB5 scale was administered by psychologists holding diplomas and trained in the use of the tool (compulsory eight-hour supervised training – on 28/06/2023 there were 10,786 licensed SB5 diagnosticians in Poland) in an office adapted for psychological examinations of children and youth. The tests were conducted individually, without the parents being present, and it took between an average of 30 minutes (youngest children and individuals with an intellectual or developmental disability to an average of 90 minutes (older children, youth).

Demographic information regarding place of residence, the gender and age of the child, as well as parents’ education were collected during an interview with the caregivers (with the child absent), done before the intelligence assessment. Data collected during the interview and the test were written down by the diagnostician in the examination protocol and the interview sheet. After the examination, the diagnostician input the demographic data and the raw test results into an on-line application, confirming consent for their use in research. Data collection focused on ensuring full anonymization of all information input into the system, hence detailed data regarding the initial referral reasons were not gathered. At this stage, in order to eliminate human error, the raw data were automatically re-calculated into standardised scores and saved together with the demographic data in a central database in an anonymised form. Neither the children nor the parents were remunerated for taking part in the study.

## Data Records

The data comprising intelligence assessment results from over four hundred thousand complete diagnoses are stored in a single table. The dataset is available in the OSF repository^34^ in two formats: xlsx, and Rda. The dataset contains 419,135 observations (each in a separate row) and 47 variables (each in a separate column).

The dataset consists of 47 variables, measured at various levels of measurement. These variables capture diverse aspects of the study’s subject matter and provide valuable information for analysis. Table S1 (see Supplementary Information document) offers a comprehensive overview of the data structure, presenting a detailed breakdown of each variable, its type of measurement, and the corresponding data points or observations.

1. **Age Group (Variable 5):** The age group is coded as ‘G’ for ‘group,’ followed by the number of years, and a sequential group number within that cohort, enclosed in parentheses. For example, ‘G10.1 (10:0-10:3)’ represents the first subgroup of ten-year-old, aged 10 years and 0 months to 10 years and 3 months.
2. **Place of Residence (Variable 6):** This variable has two categories: ‘city’ and ‘countryside.’ In Poland, ‘city’ refers to densely populated areas with limited agricultural land and a workforce primarily engaged in non-agricultural sectors. ‘Countryside’ typically represents smaller settlements with limited infrastructure and service access.
3. **Parents’ Level of Education (Variables 9, 10, and 11):** These variables have the following response categories:
  ‘Primary or Lower Secondary’: Mandatory education in Poland, spanning 8 or 9 years.
  ‘Secondary’: Additional 3 to 5 years of education, depending on the type of school, following primary education.
  ‘Vocational’: 3 years of post-primary education for specific professions.
  ‘Higher’: Includes 3 to 6 years of education beyond secondary school, at the undergraduate or postgraduate level.
4. **Raw Scores (Variables 12 to 21):** These scores represent the total points obtained by participants in tests that assess various facets of intelligence. Test responses are assessed for accuracy, completeness, and adherence to a predetermined key. Scores for individual items may range from 0 to 1 or 0 to 2 points, with higher scores signifying superior performance.
5. **Scores on a 1-19 Scale (Variables 38 to 47**): These standardized scores account for participants’ ages. The scale is designed so that the population’s average score is 10, with a standard deviation of 3. A higher score indicates better test performance. Note: Extreme scores may fall outside this theoretical range but are assigned values within the possible range.
6. **Transitional Scores on a 1–19 Scale (Variables 22 to 29):** These scores bridge the gap between the 1–19 scale and the IQ scale. They are derived by summing 1–19 scores from relevant variables. For example, ‘QR_SC_119’ is created by combining verbal and non-verbal Quantitative Reasoning scores. There are five such variables, corresponding to SB5 subtests.
7. **IQ Scale Scores (Variables 30 to 37)**: These standardized scores are directly derived from the 1–19 scale. They maintain a clear correspondence with the 1–19 scale variables. While transforming one scale into another is complex, IQ scores are designed with an average diagnostic score of 100 and a standard deviation of 15. Higher IQ scores indicate better test performance. Note: Unlike the 1–19 scale, IQ scores are not capped, allowing for any potential score.

To provide an overview of the characteristics of the study sample and the range of collected data, Tables 2 and 3 present summary statistics for selected demographic variables, categorised by age groups (Table 2) and intelligence levels (Table 3) of the observations.

**Table 2:** Observations and Descriptive Statistics for Demographic Variables by Age Group.

Variable	Age group			
	Pre-schoolers (3;00–6;11)	Early-school-age children (7;00–9;11)	Preadolescents (10;00–15;11)	Late Adolescents (16;00–18;11)
	*N (%) / M (SD)*	*N (%) / M (SD)*	*N (%) / M (SD)*	*N (%) / M (SD)*
**Gender**				
Male	53,769 (67)	73,399 (65)	12,3281 (60)	11,156 (52)
Female	26,374 (33)	39,778 (35)	81,088 (40)	10,290 (48)
**Place of Residence**				
Countryside	19,757 (25)	34,362 (31)	62,731 (31)	5,223 (25)
City	59,720 (75)	77,666 (69)	140,079 (69)	16,018 (75)
**Age**	5.41 (1.14)	8.50 (0.90)	12.34 (1.62)	17.09 (0.78)

**Table 3:** Observations and Descriptive Statistics for Demographic Variables by Intelligence Level.

Variable	Individuals with Intellectual Disabilities		Individuals with Close-to-average Intelligence		
	Moderate Intellectual Disability (IQ 35–54)	Mild Intellectual Disability (IQ 55–69)	Below-average Intelligence (IQ 70–84)	Average Intelligence (IQ 85–114)	Above-average Intelligence (IQ > 114)
	*N (%) / M (SD)*	*N (%) / M (SD)*	*N (%) / M (SD)*	*N (%) / M (SD)*	*N (%) / M (SD)*
**Gender**					
Male	15,615 (62)	31,692 (62)	63,385 (62)	139,805 (63)	11,108 (67)
Female	9,680 (38)	19,608 (38)	39,021 (38)	83,868 (37)	5,353 (33)
**Place of Residence**					
Countryside	8,738 (35)	18,437 (36)	34,653 (34)	57,745 (26)	2,500 (25)
City	16,221 (65)	32,244 (64)	66,775 (66)	164,386 (74)	13,857 (85)
**Age**	10.87 (3.99)	10.15 (3.59)	10.13 (3.38)	10.25 (3.25)	9.54 (3.59)

## Technical Validation

During the data collection process, significant attention was devoted to ensuring the high quality, reliability, and validity of measurements. To achieve these objectives, several crucial actions were undertaken:

1. Standardisation of the assessment procedure. The diagnostic process was carefully standardised to maintain consistency and uniformity across all evaluations. This step was essential to minimise any potential bias or variation in the results obtained from different diagnosticians.
2. Training of diagnosticians. Recognizing the pivotal role of diagnosticians in conducting accurate intelligence assessments, comprehensive training programs were implemented. These training sessions aimed to enhance the diagnosticians’ skills, knowledge, and understanding of the assessment tools and protocols. The training encompassed both the theoretical foundations of the SB5 and the assessment procedures using this test battery. Additionally, it addressed the specifics of conducting assessments across diverse age and clinical groups, along with ethical considerations. The training was conducted by a co-author of the Polish adaptation of the SB5, who is an expert in psychometrics, clinical psychology, developmental psychology, and psychopathology.
3. Utilisation of a validated psychometric tool. To ensure the reliability of measurements, a validated assessment instrument was employed: the Polish version of the Stanford-Binet Intelligence Scales – 5^th^ Edition. This test battery has undergone rigorous validation processes to ensure its reliability and validity for measuring intelligence in the Polish context. A detailed description of the results of the conducted validation studies is presented in the technical manual for the Polish version of the method^37^. The report specifically provides evidence of reliability (measured by internal consistency coefficients and test–retest method) and validity (e.g., correlation of SB5 results with other intelligence batteries).
4. Incorporation of reliability and standard error of measurement (SEM). To gauge the precision and consistency of the assessment results, measures of reliability and SEM were utilised. These statistical indices provided valuable insights into the consistency of the measurements, allowing researchers to make more robust interpretations.

Furthermore, the data collection context played a vital role in influencing the quality of the gathered information. Each diagnosis was conducted on a case-by-case basis, with particular attention given to the unique characteristics of the individuals being assessed. The data collection process was an integral part of a genuine examination involving individuals who were referred due to a variety of challenges, such as learning difficulties. Anonymization of data occurred as early as the stage of entering the data into the application, ensuring the privacy and confidentiality of the participants.

By prioritising measurement quality, employing rigorous assessment protocols, and considering the particularities of the context in which the data were collected, this research study aimed to maximise the credibility and robustness of its findings. The meticulous approach to data collection and analysis ultimately contributed to the integrity and trustworthiness of the research outcomes.

To validate the overall factor structure of the SB5 and support its relevance for subsequent research using this dataset, we conducted a confirmatory factor analysis (CFA). We employed a maximum likelihood estimator with the entire sample of 419,135 participants to test a two-factor model of the SB5, representing verbal and nonverbal IQ. The results from the CFA indicated an excellent fit for the two-factor model. The Comparative Fit Index (CFI) was 0.98, and the Tucker-Lewis Index (TLI) was 0.97, both suggesting a very good fit of the model to the data. The Root Mean Square Error of Approximation (RMSEA) was 0.063, with a 90% confidence interval ranging from 0.062 to 0.063, indicating an acceptable error of approximation for the model. Factor loadings ranged from 0.75 to 0.82 for verbal IQ and from 0.69 to 0.81 for nonverbal IQ, demonstrating strong associations of the tests’ scores with their respective latent factors. Furthermore, the correlation between the latent variables was notably high at 0.94, underscoring a significant overlap between verbal and nonverbal intelligence components. These findings confirm the robustness and reliability of the SB5’s two-factor structure within this large Polish sample. The strong factor loadings and high inter-factor correlation enhance our confidence in using this model to explore intellectual abilities across diverse demographic settings.

## Usage Notes

The data is available under the CC-BY Attribution 4.0 International license.

### Supplementary information

Overview of the data structure

## Data Availability

The R code containing data transformation commands is provided as a supplementary material to the database and can be found in the OSF repository^34^.
