# Supplementary material for: Intelligence Assessment of Children & Youth Benefiting from Psychological-Educational Support System in Poland
Source: Sci Data. 2024 Jul 27;11:826. doi: 10.1038/s41597-024-03663-9 (PMC11283510; doi:10.1038/s41597-024-03663-9)
Supplement: Supplementary file 1 — Overview of the data structure [file 41597_2024_3663_MOESM1_ESM.docx]

**Table S1.**

*Overview of the Data Structure*

| **No** | **Acronym** | **Missing (%)** | **Class** | **Description/remarks** | **Values** |
| --- | --- | --- | --- | --- | --- |
| 1 | id | 0.0 | numeric, integer | Identification number of diagnosis | From 9,447 to 522,762 |
| 2 | timestamp | 0.0 | character | The date the test results were entered into the system | Dates from 2018-05-20 to 2023-04-28 |
| 3 | gender | 0.0 | factor | Gender | Male, female |
| 4 | age_years | 0.0 | numeric, double | Age in years; multiplying the value by 12 gives the exact age in months at the time of diagnosis of the examined person | Real numbers from 2;0 to 18;9 |
| 5 | age_group | 0.0 | ordered, factor | Age group. The age group is coded as 'G' for 'group,' followed by the number of years, and a sequential group number within that cohort, enclosed in parentheses. For example, 'G10.1 (10:0-10:3)' represents the first subgroup of ten-year-olds, aged 10 years and 0 months to 10 years and 3 months. | From G2.1 (2:0-2:1) to G18.1 (18:00-18:11) |
| 6 | residence | 0.9 | factor | Place of residence | City, countryside |
| 7 | covid | 98.0 | factor | Confirmed COVID diagnosis before the test date. The question is optional and has been asked since halfway through the pandemic. | Yes, no |
| 8 | diagnosis | 85.8 | character | Diagnosis at the time of diagnosis. Previous, formal diagnosis made by a qualified professional. Absence of data can indicate both the absence of a formal diagnosis and the absence of information being entered on this matter. | ADD, ADHD, below average intelligence, conduct and emotional disorders, dyscalculia, dyslexia, intellectual disability, neurological disorders, other, speech disorders, traumatic brain injury |
| 9 | education_mother | 51.2 | ordered, factor | Mother's level of education was entered optionally; therefore, it is often omitted. | higher, primary or lower secondary, secondary, vocational |
| 10 | education_father | 80.6 | ordered, factor | Father's level of education was entered optionally until early 2022; therefore, it is often omitted. After this date, the data was no longer collected. | higher, primary or lower secondary, secondary, vocational |
| 11 | education_parents | 51.0 | ordered, factor | Combined, maximum parents’ education (also calculated based on partial data). | higher, primary or lower secondary, secondary, vocational |
| 12 | FR_NV_raw | 0.0 | numeric, integer | Raw result for the Nonverbal Fluid Reasoning scale | Integer numbers from 0 to 36 |
| 13 | KN_NV_raw | 0.0 | numeric, integer | Raw result for the Nonverbal Knowledge scale | Integer numbers from 0 to 30 |
| 14 | QR_NV_raw | 0.0 | numeric, integer | Raw result for the Nonverbal Quantitative Reasoning scale | Integer numbers from 0 to 30 |
| 15 | VS_NV_raw | 0.0 | numeric, integer | Raw result for the Nonverbal Visual-Spatial Processing scale | Integer numbers from 0 to 34 |
| 16 | WM_NV_raw | 0.0 | numeric, integer | Raw result for the Nonverbal Working Memory scale | Integer numbers from 0 to 34 |
| 17 | FR_VE_raw | 0.0 | numeric, integer | Raw result for the Verbal Fluid Reasoning scale | Integer numbers from 0 to 30 |
| 18 | KN_VE_raw | 0.0 | numeric, integer | Raw result for the Verbal Knowledge scale | Integer numbers from 0 to 74 |
| 19 | QR_VE_raw | 0.0 | numeric, integer | Raw result for the Verbal Quantitative reasoning scale | Integer numbers from 0 to 30 |
| 20 | VS_VE_raw | 0.0 | numeric, integer | Raw result for the Verbal Visual-Spatial Processing scale | Integer numbers from 0 to 30 |
| 21 | WM_VE_raw | 0.0 | numeric, integer | Raw result for the Verbal Working Memory scale | Integer numbers from 0 to 30 |
| 22 | NVER_SC_119 | 0.0 | numeric, integer | Summed standardised score from 1 to 19 for the Nonverbal Intelligence scale. | Integer numbers from 5 to 95 |
| 23 | VERB_SC_119 | 0.0 | numeric, integer | Summed standardised score from 1 to 19 for the Verbal Intelligence scale | Integer numbers from 5 to 95 |

**Table S1.** *(Continuation)*

| **No** | **Acronym** | **Missing (%)** | **Class** | **Description/remarks** | **Values** |
| --- | --- | --- | --- | --- | --- |
| 24 | ALL_SC_119 | 0.0 | numeric, integer | Summed standardised score from 1 to 19 for the General Intelligence scale | Integer numbers from 10 to 190 |
| 25 | FR_SC_119 | 0.0 | numeric, integer | Summed standardised score from 1 to 19 for the Fluid Reasoning scale | Integer numbers from 2 to 38 |
| 26 | KN_SC_119 | 0.0 | numeric, integer | Summed standardised score from 1 to 19 for the Knowledge scale | Integer numbers from 2 to 38 |
| 27 | QR_SC_119 | 0.0 | numeric, integer | Summed standardised score from 1 to 19 for the Quantitative Reasoning scale | Integer numbers from 2 to 38 |
| 28 | VS_SC_119 | 0.0 | numeric, integer | Summed standardised score from 1 to 19 for the Visual-Spatial Processing scale | Integer numbers from 2 to 38 |
| 29 | WM_SC_119 | 0.0 | numeric, integer | Summed standardised score from 1 to 19 for the Working Memory scale | Integer numbers from 2 to 38 |
| 30 | NVER_SC_IQ | 0.0 | numeric, integer | Nonverbal IQ | Integer numbers from 39 to 160 |
| 31 | VERB_SC_IQ | 0.0 | numeric, integer | Verbal IQ | Integer numbers from 42 to 158 |
| 32 | ALL_SC_IQ | 0.0 | numeric, integer | IQ (General) | Integer numbers from 36 to 163 |
| 33 | FR_SC_IQ | 0.0 | numeric, integer | Fluid Reasoning IQ | Integer numbers from 45 to 155 |
| 34 | KN_SC_IQ | 0.0 | numeric, integer | Knowledge IQ | Integer numbers from 48 to 151 |
| 35 | QR_SC_IQ | 0.0 | numeric, integer | Quantitative Reasoning IQ | Integer numbers from 48 to 151 |
| 36 | VS_SC_IQ | 0.0 | numeric, integer | Visual-Spatial Processing IQ | Integer numbers from 47 to 152 |
| 37 | WM_SC_IQ | 0.0 | numeric, integer | Working Memory IQ | Integer numbers from 44 to 155 |
| 38 | FR_NV_119 | 0.0 | numeric, integer | Standardised score from 1 to 19 for the Nonverbal Fluid Reasoning scale | Integer numbers from 1 to 19 |
| 39 | KN_NV_119 | 0.0 | numeric, integer | Standardised score from 1 to 19 for the Nonverbal Knowledge scale | Integer numbers from 1 to 19 |
| 40 | QR_NV_119 | 0.0 | numeric, integer | Standardised score from 1 to 19 for the Nonverbal Quantitative Reasoning scale | Integer numbers from 1 to 19 |
| 41 | VS_NV_119 | 0.0 | numeric, integer | Standardised score from 1 to 19 for the Nonverbal Visual-Spatial Processing scale | Integer numbers from 1 to 19 |
| 42 | WM_NV_119 | 0.0 | numeric, integer | Standardised score from 1 to 19 for the Nonverbal Working Memory scale | Integer numbers from 1 to 19 |
| 43 | FR_VE_119 | 0.0 | numeric, integer | Standardised score from 1 to 19 for the Verbal Fluid Reasoning scale | Integer numbers from 1 to 19 |
| 44 | KN_VE_119 | 0.0 | numeric, integer | Standardised score from 1 to 19 for the Verbal Knowledge scale | Integer numbers from 1 to 19 |
| 45 | QR_VE_119 | 0.0 | numeric, integer | Standardised score from 1 to 19 for the Verbal Quantitative Reasoning scale | Integer numbers from 1 to 19 |
| 46 | VS_VE_119 | 0.0 | numeric, integer | Standardised score from 1 to 19 for the Verbal Visual-Spatial Processing scale | Integer numbers from 1 to 19 |
| 47 | WM_VE_119 | 0.0 | numeric, integer | Standardised score from 1 to 19 for the Verbal Working Memory scale | Integer numbers from 1 to 19 |
